# Unraveling the Microbial Interactions and Metabolic Potentials in Pre- and Post-treated Sludge from a Wastewater Treatment Plant Using Metagenomic Studies

**DOI:** 10.3389/fmicb.2017.01382

**Published:** 2017-07-19

**Authors:** Chandni Sidhu, Surendra Vikram, Anil Kumar Pinnaka

**Affiliations:** ^1^Microbial Type Culture Collection and Gene Bank, Council of Scientific and Industrial Research-Institute of Microbial Technology Chandigarh, India; ^2^Centre for Microbial Ecology and Genomics, Department of Genetics, University of Pretoria Pretoria, South Africa

**Keywords:** carbon mineralization, virulence, metagenomes, oxygenases, anaerobic digestion

## Abstract

Sewage waste represents an ecosystem of complex and interactive microbial consortia which proliferate with different kinetics according to their individual genetic as well as metabolic potential. We performed metagenomic shotgun sequencing on Ion-Torrent platform, to explore the microbial community structure, their biological interactions and associated functional capacity of pre-treated/raw sludge (RS) and post-treated/dried sludge (DS) of wastewater treatment plant. Bacterial phylotypes belonging to *Epsilonproteobacteria* (∼45.80%) dominated the RS with relatively few Archaea (∼1.94%) whereas DS has the dominance of *beta*- (30.23%) and *delta*- (13.38%) classes of *Proteobacteria* with relatively greater abundance of *Archaea* (∼7.18%). In particular, *Epsilonproteobacteria* appears as a primary energy source in RS and sulfur-reducing bacteria with methanogens seems to be in the potential syntrophic association in DS. These interactions could be ultimately responsible for carrying out amino-acid degradation, aromatic compound degradation and degradation of propionate and butyrate in DS. Our data also reveal the presence of key genes in the sludge microbial community responsible for degradation of polycyclic aromatic hydrocarbons. Potential pathogenic microbes and genes for the virulence factors were found to be relatively abundant in RS which clearly reflect the necessity of treatment of RS. After treatment, potential pathogens load was reduced, indicating the sludge hygienisation in DS. Additionally, the interactions found in this study would reveal the biological and environmental cooperation among microbial communities for domestic wastewater treatment.

## Introduction

Sewage waste is a heterogeneous mixture of various known and unknown saprophytic, pathogenic and non-pathogenic microbes such as bacteria, viruses, protozoa, and fungi. Additionally, the sewage waste also contains organic materials including hazardous chemicals (e.g., pesticides, detergents, fats, oils, solvents, phenol and pharmaceutical wastes) which can give rise to potential health hazards and therefore, must be subjected to biological, chemical, and thermal treatments to reduce the load of pathogens and toxic compounds (Yu and Zhang, 2012). Wastewater treatment plants (WWTPs) make use of biological processes, which involves the use of complex microbial consortia consisting of *Euryarchaeota*, fungi, and bacteria in the form of activated sludge for the removal of organic wastes and nutrients to reduce the hazardous health risk associated with it (Cai et al., 2014; Ju et al., 2014).

Microbial dynamics studies and profiling of these functional bacteria in various ecosystems (soil, activated sludge and sediments) have already been conducted by using both culture-dependent and PCR-based (16S rRNA gene amplicon sequencing) culture-independent methods (McLellan et al., 2010; Yu and Zhang, 2012). Limited cultivability of microbes, PCR biases and limitation of primer efficiencies limits the application of these methods conducting the microbial ecology studies (Handelsman, 2004; Riesenfeld et al., 2004; Blow, 2008). Particularly, these methods solely explore the bacterial diversity with no functional relevance. However, the shotgun metagenomic approach based on direct environmental DNA sequencing makes the exploration of hidden microbial communities structure and functional assumptions more reliable and informative (Handelsman, 2004). Importantly, the fundamental goal of microbial ecology is to understand the microbial interactions (Gilbert et al., 2012). The strikingly diverse mass of microbial species within a microbiome leads to dynamic spatio-temporal interactions constituting cooperation, competition, and communication among themselves (Hibbing et al., 2010).

Wastewater treatment plant based on Upflow Anaerobic Sludge Blanket (UASB) technology is used extensively as the major type of biological treatment around the world (Ahring, 2003). It is the most successful and commercialized anaerobic digestion reactor configuration and represents an ecosystem of diverse structural as well as functional interactions among microbes. The stage of biological process (activated sludge) has been studied extensively by many research groups over the past years (Kanazawa, 1987; McLellan et al., 2010; Yu and Zhang, 2012; Zhang et al., 2013; Jadeja et al., 2014; Ju et al., 2014; Yadav et al., 2015), but very few studies have been conducted so far which shed light on the functional potential of sludge microorganisms during pre- and post-stages of biological treatments (Luo et al., 2016; Mei et al., 2016). The stage prior to biological treatment is called raw sludge (RS) containing the pre-treated primary waste directly from the influent pipes, whereas the remains of sludge after biological digestion and during the air-drying stage is designated as dried sludge (DS).

Detoxification of DS could be of major concern as these residual solids are directly discharged into the environment and contain a majority of prokaryotic biomass (Burch et al., 2013). It could be targeted to remove the antibiotic resistance/virulence genes by reducing the pathogenic load of WWTPs. The present study reports the limitation of air-drying of sewage waste after biological treatment. Also, RS is considered to be a habitat of various pathogenic microbes that may threaten public health (Varela and Manaia, 2013). This study would help in determining the fate of these pathogens and associated virulence genes during the anaerobic treatment process.

To fulfill our prime objective, we employed high throughput shotgun sequencing using Ion-torrent NGS platform to characterize and dissect the complete and detailed phylogenetic and functional microbial structures and to visualize the complete picture of biological interactions taking place at both pre- and post-treated sludge stages of WWTP. These results could help to understand the previously unknown metabolic and functional potential of pre- and post-stages of activated sludge. In addition, the study could help to deduce the possible metabolic pathways associated with each stage and role of microbial interactions to perform the degradation and other metabolic activities.

## Materials and Methods

### Site Description and Sample Collection

Wastewater treatment plant, Raipur Kalan, (Longitude: 30°41′12.335′′N; Latitude: 76°49′20.497′′E) located at a distance of 6 km from Chandigarh, India, was used as sampling source to investigate both the microbial community structure and associated functional potential. It is based upon UASB technology. Two different sites of WWTP were used for sample collection. First, the pre-treated sludge (RS) from the primary reactor was collected and then the sludge from drying beds (DS) was taken. Samples were brought to the laboratory under cold conditions and processed immediately for metagenomic DNA isolation.

### Metagenomic DNA Extractions

Metagenomic DNA was isolated from all the sludge samples using CTAB extraction method (Dounghatai et al., 2012). Briefly, 500 μL of sludge sample was taken in a 1.5 mL microcentrifuge tube and centrifuged at 21,913 × *g* for 10 min. Pellet was lysed by adding an equal volume of sterile glass beads of size < 150 microns (Sigma) and 500 μL of CTAB extraction buffer (1 M Tris-Cl, pH 8.0, 5 M NaCl, 0.5 M EDTA, 2% CTAB). The mixture was vortexed for 1 min and extracted using phenol:chloroform:isoamylalcohol (25:24:1). The resulting suspension was vortexed for 1 min and frozen for 1 min. Aqueous phase was separated by centrifugation at 21,913 × *g* for 10 min at 4°C. Phenol was removed by mixing 500 μL chloroform:isoamylalcohol (24:1) and centrifugation at 21,913 × *g* for 10 min at 4°C. DNA was precipitated with 0.1 volume of 3 M filter-sterilized sodium acetate and 0.6 volume of isopropanol at 20°C for 30 min. DNA pellet was washed twice with chilled 70% ethanol, air-dried for 20 min and re-suspended in 30 μL low TE buffer. DNA was quantified and checked for purity at A260/280 nm and A260/230 nm by Nanodrop 1000 spectrophotometer (Thermo Scientific), prior to storage at -20°C. The quality of DNA was also checked by 0.8% agarose gel electrophoresis.

### Metagenomic Analysis by Shotgun Sequencing

Metagenomic DNA isolated from all the sludge samples was accessed for its concentration using Qubit^®^2.0 Fluorometer (Invitrogen, Life Technologies) before proceeding to shotgun library preparation. Briefly, DNA was sheared using Bioruptor^®^UCD 200 sonication system (Diagenode). The fragmented DNA was end-repaired and ligated to Ion adapters according to manufacturer’s protocol. DNA template (200 bp chemistry) was amplified on Ion Sphere Particles (ISPs) using Ion OneTouch Instrument. Positive templates were enriched and sequenced on 318 microchip using Ion Torrent Personal Genome Machine (Life Technologies, United States). Data files were exported as *sff* and *fastq* format after sequencing.

### Bioinformatic Analysis of Sequences Retrieved in All Datasets

The quality of the reads was analyzed using the Prinseq lite v0.20.4 (Schmieder and Edwards, 2011). Reads having mean phred quality score < 20, ambiguous bases (N’s) and ≤35 bp were removed. Also, the reads were trimmed from the right (5′ end) having bases phred score < 20. The reads for all the sludge samples were sub-sampled for the lowest number of the reads for RS sample.

The high quality sub-sampled reads from each sample were used for the Blastx (at e-value 1e-3) analysis using diamond program (Buchfink et al., 2015) against NCBI-NR database. The resulting Blastx tab files were analyzed with MEGAN v5.10.6 for the taxonomic and functional analysis. The KEGG orthology and SEED subsystems were analyzed for the metagenomes. The taxonomic and functional comparison of datasets was executed by MEGAN v5.10.6 using absolute count parameters (Huson et al., 2007). Statistical Analysis of Metagenomic Profiles (STAMP v2.1.3) was also used to compare the taxonomic as well as functional (SEED, COG, KEGG) abundance. Two-sided Fischer’s exact test with a CI (Confidence interval) of 0.95 was used to identify the significant changes in both the datasets. Story FDR (False Discovery Rate) with the corrected *p*-value < 0.05 was used to correct the comparisons.

### PCR Amplification, Cloning and Community Structure Analysis

Purified DNA from sludge was used to amplify methanogen 16S rRNA gene using methanogen-specific primers: Met 83F-ACKGCTCAGTAACAC and Met 1340R-CGGTGTGTGCAAGGAG (Banning et al., 2005), and PCR amplification was carried out in triplicates. PCR products were pooled, purified by gel extraction using QIAquick gel Extraction Kit (Qiagen) and cloned using CloneJET^TM^ PCR Cloning Kit (Thermo Scientific) using manufacturer’s instructions. Forty random clones from methanogen 16S rRNA gene clone library were picked and checked for gene insertion using vector-specific primers. Gene positive clones were then sequenced using ABI 3130xl Genetic Analyzer, A16 capillary machine (Applied Biosystems, Carlsbad, CA, United States). Phylogenetic analysis of gene sequences and tree construction was done by MEGA6 software (Tamura et al., 2013) using Neighbor-joining method (Saitou and Nei, 1987). Bootstrap analyses for 500 resamplings were performed to provide confidence estimates for tree topologies (Felsenstein, 1985).

### Nucleotide Sequence Accession Numbers

The 16S rRNA gene sequences of all methanogen clone isolates have been deposited in GenBank, EMBL, and DDBJ nucleotide sequence databases and assigned accession no. KT963468-KT963507. Raw read data of both RS and DS metagenomes were deposited at NCBI under the Bioproject ID: PRJNA304667 and SRA accession numbers for RS and DS samples are SRR2968850 and SRR2968863, respectively.

## Results

### General Characteristics and Sequencing Data

General parameters of WWTP, Raipur Kalan, as analyzed previously, were given in Supplementary Table S1. For sequencing, we have used 1 Gb depth for each RS and DS with average read length of 176 ± 58 bp and 179 ± 59 bp, respectively. Ion PGM-200 sequencing of metagenomic DNA of both samples generated a total of 11,707,454 reads sequence data. The quality filter of sequence reads resulted in 11,142,677 reads sequence data (Supplementary Table S2). The reads were sub-sampled based on lowest number of reads in RS (5,229,283) and subjected to analysis.

### Comparison of Microbial Community Structure of Raw Sludge and Dried Sludge

**FIGURE 1:**
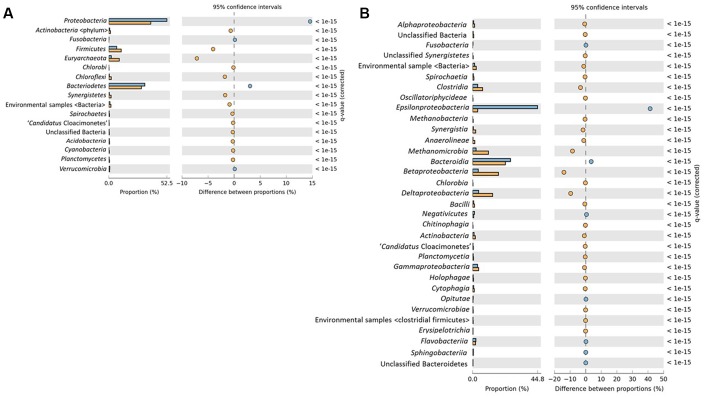
Significant differences in microbial communities of raw sludge (RS) (blue) and dried sludge (DS) (orange) at **(A)** Phylum and **(B)** Class level distribution. Significance was determined using two-sided Fischer’s exact test with a CI (Confidence interval) of 0.95 and Story False Discovery Rate (FDR) with the corrected *p*-value < 0.05 was used to correct the multiple comparisons.

Taxonomic profiling of both sludge stages was done by analyzing the reads using two different approaches; the ribosomal small subunit (SSU) using Metaxa2 (Bengtsson-Palme et al., 2015) and MEGAN v5.10.6 (Huson et al., 2007) (Supplementary Figure S1). Additionally, STAMP was used for the statistical verification of the taxonomic and functional data. Results from all approaches were mostly similar and data analysis showed dominance of the domain *Bacteria* (97.9%) in RS whereas DS has relatively less abundance (92.65%). Interestingly, *Archaea* (7.18%) has been found to be present in greater abundance in DS as compared to RS which constitutes only 1.94% of the total community found. At phylum level, both the samples were dominated by *Proteobacteria* (RS 53.21%; DS 38.8%), *Bacteroidetes* (RS 32.9%; DS 30.2%), and *Firmicutes* (RS 7.2%; DS 11.6%) whereas other phyla; *Actinobacteria* (RS 0.84%; DS 1.5%), *Chloroflexi* (RS 0.48%; DS 2.31%), *Synergistetes* (RS 0.45%; DS 2.2%), and *Verrucomicrobia* (RS 0.93%; DS 0.83%) were present as relatively minor contributors to total microbial community (**Figure 1A**). STAMP results showed the high correlation of both samples at phylum level (*R*^2^ = 0.95).

Class level distribution showed significant variability among various classes of *Proteobacteria*. *Epsilonproteobacteria* have been identified as the major component of microbial communities in RS dataset whereas DS was found to be dominated by *Beta*- and *Delta*-classes of *Proteobacteria* followed by methanogens (**Figure 1B**).

Species level distribution using STAMP showed the dominance of different divisions of microbial communities in both datasets. DS showed the dominance of SRBs along with syntrophs and methanogens whereas RS showed a high abundance of *Sulfurospirillum* genus, belonging to *Epsilonproteobacteria* (Supplementary Figure S2).

Methanogens were found as key players in anaerobic DS. In this study, the diversity and phylogeny of methanogens present in both RS and DS have been checked using methanogen-specific primers (Banning et al., 2005), but amplification product could be found only in DS. Forty random clones from clone library were sequenced for elucidating diversity and evolutionary relationships among methanogens present in DS. Results from EzTaxon were used to construct the phylogenetic tree (Supplementary Figure S3A). EzTaxon mapping showed that most of the sequences (48%) were phylogenetically associated with *Methanosaeta* genus within acetoclastic *Methanosarcinales*. Interestingly, 17% of the sequence types were found to be associated with genus *Thermogymnomonas* which were not annotated by shotgun sequencing. The clone distribution was 19, 7, 6, 3, 3, and 2 for *Methanosaeta* (order *Methanosarcinales*), *Thermogymnomonas* (order *Thermoplasmatales*), *Methanolinea*, *Methanoregula*, *Methanobacterium*, and *Methanospirillum* (order *Methanomicrobiales*), respectively. The abundance of *Methanosaeta* and *Thermogymnomonas* has previously been found to be among the dominating archaeal communities that contribute majorly for granular sludge in drying beds (Nishio and Nakashimada, 2004). Methanogens diversity obtained by shotgun sequencing was also compared with sequences obtained from clone library (Supplementary Figure S3B). DS dataset showed the high abundance of *Methanosaeta* (43.10%) (acetoclastic methanogens) followed by *Methanospirillum* (33.49%), *Methanoregula* (12.24%) (Hydrogenotrophic methanogens), and *Methanolinea* (7.22%) and *Methanobacterium* (3.95%). Moreover, the methylotrophic methanogens (e.g., *Methanococcoides, Methanohalophilus*) were not found in DS.

### Functional Profiling of Microbial Communities Present in Raw Sludge and Dried Sludge

To understand the functional potential of bacterial diversity in both datasets, we used MEGAN v5.10.6 to assign functions to the reads. MEGAN analysis showed that of a total of 3,358,226 reads, an estimated 924,343 were successfully assigned to the KEGG orthology (KO numbers) by MEGAN, whereas 777,955 were annotated to biological SEED subsystem proteins using NCBI-NR protein database.

**FIGURE 2:**
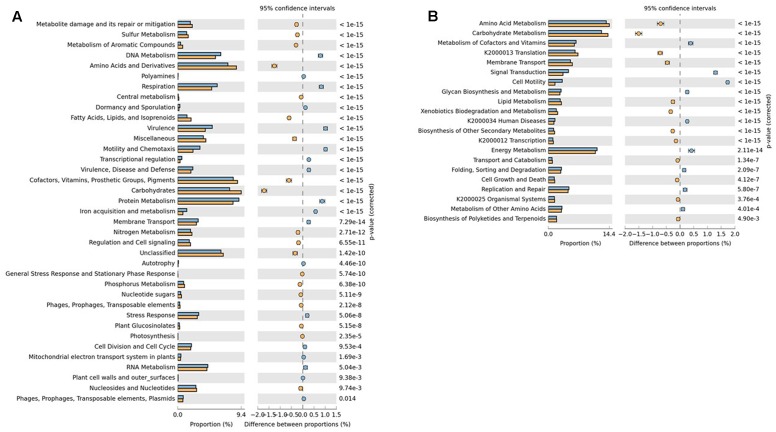
Significant functional abundance differences in **(A)** SEED and **(B)** KEGG subsystems in RS (blue) and DS (orange) datasets. Significance was determined using two-sided Fischer’s exact test with a CI (Confidence interval) of 0.95 and Story FDR with the corrected *p*-value < 0.05 was used to correct the multiple comparisons.

Comparison of SEED subsystems using STAMP revealed the genes for virulence, motility and chemotaxis, DNA metabolism, protein metabolism and respiration were significantly over-represented in RS whereas DS was dominated by genes related to amino-acids and derivatives, carbohydrate metabolism and fatty acids, lipids and isoprenoids metabolism, which may be due to the degradation of organic matter in drying sludge blankets (**Figure 2A**). KEGG analysis showed that pathways related to signal transduction, cell motility and virulence were over-represented in RS whereas DS was found to be enriched in amino-acid and carbohydrate metabolism pathways (**Figure 2B**). As RS is pre-treated waste mainly containing human excreta, the presence of genes related to the subsystem virulence and other putative virulence factors, e.g., flagellar proteins, genes for motility and chemotaxis were prevalent. The genes over-represented in DS have already been reported in the anaerobic sludge metagenomes (Cai et al., 2016).

SEED subsystems classification of RS suggests that pathways related to *Campylobacter* iron metabolism, iron acquisition in *Vibrio*, respiration/Human Gut Microbes (HGM), cobalt-zinc-cadmium resistance, multidrug resistance efflux pumps and mycobacterial genes related to *Mmpl*6 membrane protein cluster, virulence operon, Hsp225 (pathogenesis) were highly abundant. Raw sewage represents the untreated mixture of human and animal waste and is usually associated with waterborne pathogens that can cause serious health problems and are well-known as human and animal pathogens. Most of the above genes were mapped to *Campylobacter* species which indicates the need for the treatment of RS to reduce their abundance counts. On the other side, DS dataset was found to be abundant with the pathways like acetoin-butanediol metabolism, fermentation, methanogenesis, methylcitrate cycle, fatty acid degradation regulons, isoprenoid biosynthesis, polyhydroxybutyrate metabolism, nitrogen fixation and ammonia assimilation.

**FIGURE 3:**
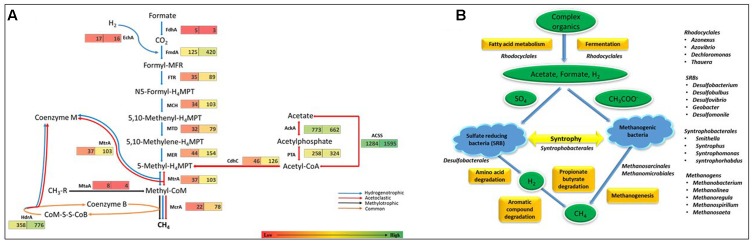
**(A)** Number of genes involved in the relevant methanogenesis pathways in both RS and DS datasets. Number in boxes indicates the reads assigned at each level. The three known pathways involved in methanogenesis are colored differentially. The hydrogenotrophic pathway is marked in blue arrows, the acetoclastic pathway is shown in red arrows, and the methylotrophic pathway is presented in black arrows. The pathway common to all is represented by brown headed arrows. FdhA, glutathione-independent formaldehyde dehydrogenase; EchA, hydrogenase subunit A; FmdA, formylmethanofuran dehydrogenase subunit A; FTR, formylmethanofuran-tetrahydromethanopterin *N*-formyltransferase; MCH, methenyltetrahydromethanopterin cyclohydrolase; MTD, methylenetetrahydromethanopterin dehydrogenase; MER, coenzyme F420-dependent N5, N10-methenyltetrahydromethanopterin reductase; MtrA, tetrahydromethanopterin *S*-methyltransferase; MtaA, [methyl-Co(III) methanol-specific corrinoid protein]:coenzyme M methyltransferase; McrA, methyl-coenzyme M reductase alpha subunit; AckA, acetate kinase; ACSS, acetyl-CoA synthetase; PTA, phosphate acetyltransferase; HdrA, heterodisulfide reductase subunit A; CdhC, acetyl-CoA decarbonylase/synthase complex subunit beta. **(B)** Schematic representation of anaerobic carbon mineralization in DS with the microbial communities involved at each step.

As methanogenesis pathways are highly prevalent in previously reported anaerobic sludge metagenomes, KEGG orthology analysis was used to elucidate methanogenesis in DS. Three types of methanogenesis pathways have been reported in archaea, namely, acetoclastic, hydrogenotrophic and methylotrophic pathways (Liu and Whitman, 2008). Hydrogenotrophic pathway includes the successive reduction of CO_2_ to methane through formyl, methylene, and methyl levels. The methyl group is then transferred to coenzyme M, forming methyl-CoM which then reduced to methane using methyl coenzyme M reductase (Mcr) at the final step (blue line in **Figure 3A**). The acetoclastic pathway includes the conversion of acetate to acetyl-CoA by utilizing phosphotransacetylase (PTA). Acetyl-CoA is then converted to a methyl group and subsequently to methane with the help of enzymes Cdh, Mtr, and Mcr (red line in **Figure 3A**). The methylotrophic pathway includes the transfer of methyl-groups from methylated compounds or methane to a methanol-specific corrinoid protein (black line in **Figure 3A**) forming methyl-CoM which then enters the methanogenesis pathway and reduces to methane via Mcr reductase (Guo et al., 2015).

KEGG reads assigned to the category “Methanogenesis” were analyzed and results showed the abundance of genes for acetoclastic methanogenesis (80.9%) (EC 2.1.1.86, EC 2.8.4.1, EC 2.3.1.169, EC 2.7.2.1, EC 2.3.1.8, and EC 6.2.1.1) followed by hydrogenotrophic pathways (EC 1.2.1.46, EC 1.2.99.5, EC 2.3.1.101, EC 3.5.4.27, EC 1.5.98.1, and EC 1.5.99.11) and negligible in case of methylotrophic pathway suggesting acetoclastic methanogenesis as the dominant pathway. Genes for methanogenesis were also found in RS, but found to be relatively low in number, suggesting methanogenesis as the key process only in anaerobic digestion in DS (**Figure 3A**).

## Discussion

Wastewater treatment plant based on UASB technology has been widely used to decompose organic and human waste (Yang et al., 2014). Studies that decipher the community composition of anaerobic sludge has also been conducted by many research groups across the world (Guo et al., 2015; Sun et al., 2015; Ferrentino et al., 2016; Ju et al., 2016) but the studies conducting the detailed investigation of taxonomic as well as functional composition of pre- and post-treated anaerobic sludge are very few. Researchers have used different sequencing depths ranging from 0.1 GB to 3.3 GB to study functional composition using the Ion-torrent platform (Joshi et al., 2014; Patel et al., 2014; Rua et al., 2015; Hinsu et al., 2017; Motooka et al., 2017). Here, we employed ∼1 GB sequencing depth for shotgun metagenomic sequencing to inspect the microbial shifts in pre- and post-treated sludge along with their functional alterations.

In a study conducted by Cai et al. (2016), the presence of functional genes in an anaerobic system was related to its substrate availability. They also revealed the abundance of genes related to fatty acid and lipid metabolism in anaerobic WWTP having human excreta as a substrate (Cai et al., 2016). On the contrary, our analysis showed the abundance of genes of subsystem “amino-acids and derivatives” and “carbohydrates metabolism”. This may be due to low lipid content and high carbohydrate content in post-treated sewage sludge. Change in community structure in both datasets suggested the enrichment of particular group of microbes to utilize their feedstocks as carbon and energy source.

### Prevalence of Virulence Factors in Raw Sludge

From a public health prospective, it is necessary to treat RS as it may pose a significant health issue if not treated properly. The composition of pathogens in RS depends on the type of waste under treatment (Rais, 1989). Pathogenic microbes that are reportedly found in untreated sludge from human excreta include *Salmonella* spp., *Shigella* spp., *Escherichia coli*, *Clostridium* spp., *V. cholerae*, *Mycobacterium* spp., *Campylobacter* spp., etc., MEGAN analysis also showed the high relative abundance of these species in RS (Dumontet et al., 2001). After treatment, the pathogenic load gets reduced as observed in DS dataset. During drying of sludge, the water content of the system is critically reduced to <10% (Strauch et al., 2003) which inhibits the re-growth of many pathogens resulting in DS hygienisation. The abundance of pathogens present in RS indirectly reveals the current health status of the local population (Arthurson, 2008; Varela and Manaia, 2013). The abundance of *Mycobacterium* virulence operons and regulons, chaperones required for the pathogenesis (Hsp225), Mmpl6 membrane protein cluster and multidrug resistance efflux pumps uncover the prevalence of infectious diseases in the local population.

Moreover, iron acquisition by pathogens is related to bacterial diseases. Bacteria need iron for its metabolism and transport systems, deprivation of which eliminates bacterial virulence (Cornelis and Andrews, 2010). Gram-negative bacteria contains TonB-dependent iron uptake pathways to internalize heme and therefore, to maintain its pathogenicity (Wooldridge and Williams, 1993). SEED analysis of RS data showed the over-representation of genes linked to iron acquisition in *Campylobacter*, *Vibrio*, and TonB-transport systems. Moreover, bacterial pathogenicity is also described in terms of the route of invasion into the host cell (Duan et al., 2013). Flagella are the essential structures in many bacteria required for the motility and chemotaxis. Studies suggest that flagellum-mediated motility serves as a virulence factor in several gram-negative pathogens (Haiko and Westerlund-Wikstrom, 2013). Also, flagellum-mediated motility is responsible for pathogen-host interactions and thus provides flagella a role in pathogenesis. The number of reads belonging to flagellar movement and chemotaxis were relatively abundant in RS which illustrate the need to cut down the pathogenicity levels. Signal transduction networks are one of the essential factors that regulate the virulence factors in pathogenic bacteria and make them competent to survive in the adverse conditions (Gross, 1993). Reads mapped to signal transduction genes were also found to be relatively abundant in RS which once again gives us insights into the virulence of RS.

Although pathogenic and virulence counts have found to be declined in DS, but it was not fully eradicated. This may be because drying beds promote the growth of pathogenic bacteria when absorbs water from the environment and also some microorganisms are reluctant to adverse conditions (Strauch et al., 2003). Since thermally DS is more reliable in terms of virulence reduction, its metagenomic study would be more informative. Moreover, metagenomic studies by isolating phage-DNA instead of whole-community DNA would gain more information regarding pathogenicity count of pre- and post-treated sludge (Edwards and Rohwer, 2005; Withey et al., 2005; Hayes et al., 2017).

### Anaerobic Carbon Mineralization in Dried Sludge

Interestingly, sulfur-reducing bacteria (SRB) belonging to genera *Desulfobacterium*, *Desulfobulbus*, *Desulfovibrio*, and *Geobacter* along with syntrophic bacteria containing genera *Desulfomonile*, *Smithella*, *Syntrophus*, *Syntrophobacteria*, and *Syntrophorhabdus* exists as a leading group in DS. In sulfate-rich environments, both SRB and methanogenic archaea compete for common substrates, such as H_2,_ formate, and acetate (Dar et al., 2008; Muyzer and Stams, 2008). But depending on prevailing environmental conditions, SRB perform metabolic functions actively by growing in syntrophy with methanogens (Morris et al., 2013; Ali Shah et al., 2014). Our results also suggest the predominance of both in DS. Thus, during anoxic conditions, H_2_ produced by SRB could act as an electron shuttle for methanogens. Methanogens utilize H_2_ as an electron donor to produce CH_4_ in the presence of CO_2_ that acts as an electron acceptor thus, maintaining syntrophic associations (Raskin et al., 1996; Plugge et al., 2011). Also, in methanogenic environments, hidden sulfur cycle contributes up to 36–50% of anaerobic carbon mineralization (Pester et al., 2012). These types of microbial interactions have already been found to be widespread in methanogenic environments, such as represented by DS. Further, wastewaters have been known to be enriched with large amounts of lipids and long chain fatty acids, thereby, supports the growth of syntrophic communities (Hatamoto et al., 2007). Also, degradation of organic matter by SRBs lead to the formation of metabolites like succinate and formate which in turn produces H_2._ Therefore, we compared the taxonomic distribution for KEGG orthology subsystem “Fatty acid metabolism” for DS and we found that *Deltaproteobacteria* contributes majorly (52.44%) followed by *Betaproteobacteria* (35.07%). Among *Deltaproteobacteria*, *Syntrophobacterales* contributes 49.02%, out of which 89.88% were *Smithella* spp., therefore, we suggest the syntrophic degradation of long chain fatty acids in DS (**Figure 3B**). Also, the abundance of *Smithella* spp. among *Syntrophobacterales* indicates syntrophic degradation of propionate and butyrate (Stams and Dong, 1995; Liu et al., 1999; de Bok et al., 2001). The comparison of abundance of genes involved in fatty acid metabolism pathway in both datasets has been done in KEGG orthology analysis and results suggest the relatively high abundance of fatty acid metabolic pathway genes in DS, thereby, supporting our analysis. On the basis of reads assigned to each subsystem involved in carbon mineralization, we reconstructed the key processes of DS. Basically, fatty acid metabolism and fermentation are the processes that are involved in the conversion of complex organics (carbohydrates, proteins, lipids) into the simpler ones (acetate, formate, H_2_) and microbial communities belonging to order *Rhodocyclales* seems to be actively involved in it. Association of SRBs, methanogens and syntrophic bacteria is responsible for carrying out degradation of amino-acids, aromatic compounds and propionate and butyrate which ultimately leads to the formation of CH_4_ (**Figure 3B**).

Functionally, methanogenesis, fermentation and carbon fixation subsystems are linked to each other and the taxa involved in these pathways are specialized in carrying out microbial metabolism using C1 compounds and/or acetate during anoxic environments (Kotsyurbenko, 2005). Therefore, number of reads account for these subsystems were compared in both sludge stages and it has been found that there is a progressive increase in annotated genes of these subsystems from RS (25.5%) to DS (43.2%). It shows that in DS, anoxic conditions could lead to the enrichment of *Euryarchaeota* which further could enhance the anaerobic microbial metabolism for efficient biotransformation of organic compounds.

### Biodegradation of Organic Matter

Since sewage sludge represents complex microbial consortia associated with biodegradation processes, we tended to explore the functional pathways of biodegradation and community structure associated with it.

As oxygenases play a crucial role in biodegradation process (Fetzner, 2012) and are the fundamental enzymes involved in degradation of xenobiotics (van Hellemond et al., 2007), it would be interesting to study the variation in oxygenases associated with each dataset. SEED analysis for the subsystem “Metabolism of Aromatic Compounds” showed the abundance of 42 diverse oxygenases belonging to 17 different degradation pathways in both datasets revealing the high concentration of aromatic compounds in sewage.

Since DS is representing the anaerobic environment, we tended to study the presence of anaerobic degradation functional markers in it. During anoxic conditions, a variety of aromatic compounds get first converted into few central intermediates (e.g., benzoyl-CoA, hydroxybenzoyl-CoA), which are further reduced to acyclic products. The oxidation of these acyclic products ultimately leads to the formation of acetyl-CoA (Egland and Harwood, 2000; Fuchs et al., 2011). These transformations are mediated by different peripheral pathways of aromatic degradation (Carmona et al., 2009). Benzoyl-CoA reductase (BCR) is an important enzyme that cleaves the aromatic ring of benzoyl-CoA, by the modified β-oxidation pathway of fatty acid metabolism (Heider and Fuchs, 1997; Lahme et al., 2012). Along with it, enoyl-CoA hydratase and 4-hydroxybenzoyl-CoA reductase are the other enzymes involved in BCR mediated anaerobic degradation of benzoate (Carmona et al., 2009) and phenolic compounds (Fuchs, 2008). Distribution comparison of these marker genes in both datasets by KEGG categories showed the high relative abundance of these markers in DS. SEED subsystem analysis also showed the dominance of these markers in DS only, suggesting anaerobic degradation of aromatic compounds in DS.

Furthermore, KEGG pathways which showed greater abundance in DS includes benzoate degradation, chlorocyclohexane and chlorobenzene degradation, ethylbenzene, styrene, atrazine, caprolactam, and naphthalene degradation pathways (Supplementary Figure S4). Out of these, a maximum number of annotated reads (2,264) were assigned for benzoate degradation which suggests the degradation of benzene ring via anaerobic pathways. The extensive use of insecticides, pesticides, and organic solvents has led to a widespread release of many chemically stable and recalcitrant compounds (Seo et al., 2009). It is known that chemical degradation of these compounds is limited under aerobic conditions which eventually leads to its accumulation (Jothimani et al., 2003). But during anoxic conditions, the response of microbial consortia to these anthropogenic compounds leads to different types of microbial associations (McInerney et al., 2009). These associations could help the microorganisms to use the end products of degradation reactions as an alternative electron source in anaerobic conditions (Philipp and Schink, 2012; Morris et al., 2013). Taxonomy assignment of these KEGG pathways showed the abundance of *Betaproteobacteria* in chlorocyclohexane and chlorobenzene, caprolactam, atrazine and styrene degradation pathways, whereas ethylbenzene degradation pathway showed the abundance of both syntrophs and *Betaproteobacteria*, indicating their possible association for degradation of end products as an alternative source of organic carbon (Morris et al., 2013). Microbial communities involved in benzoate degradation pathway showed the abundance of *Beta*- and *Deltaproteobacteria* followed by methanogens, *Firmicutes* and *Chloroflexi*, whereas taxa assignment to naphthalene degradation pathway showed the abundance of *Gamma*- and *Deltaproteobacteria*.

Raw sludge has the abundance of reads responsible for chloroalkane/chloroalkene degradation, toluene and bisphenol degradation pathways. Chloroalkane/chloroalkenes are among the most widespread groundwater contaminants (Mattes et al., 2010; Maphosa et al., 2012) and this study suggests that their maximum removal could occur in the RS itself. KEGG taxonomy showed the genera of *Sulfurospirillum* to be highly active and abundant in all these pathways of RS suggesting the ability of *Epsilonproteobacteria* in degradation of xenobiotic compounds.

## Conclusion

This study provides comparative and detailed analysis of the taxonomic and functional potential of microbial consortia associated with pre- and post-stages of sludge treatment process. Our analysis has shown the predominance of different microbes associated with each sludge stage and performing functions related to carbon mineralization and biodegradation of organic matter. Although, we have found the reduction in pathogenic load and virulence genes count in DS, there was no drastic depletion, therefore, an improved method for treatment of residual solids is required.

## Author Contributions

CS designed the experiments, participated in the bioinformatic analyses and manuscript writing. CS and SV performed the bioinformatics analyses and manuscript revision process. AP designed the study, participated in coordination and helped to draft the manuscript. All authors read and approved the final manuscript.

## Conflict of Interest Statement

The authors declare that the research was conducted in the absence of any commercial or financial relationships that could be construed as a potential conflict of interest.
