# Supplementary material for: Unraveling the Microbial Interactions and Metabolic Potentials in Pre- and Post-treated Sludge from a Wastewater Treatment Plant Using Metagenomic Studies
Source: Front Microbiol. 2017 Jul 19;8:1382. doi: 10.3389/fmicb.2017.01382 (PMC5515832; doi:10.3389/fmicb.2017.01382)
Supplement: Supplementary file 1 [file Data_Sheet_1.doc]

**Supplementary Materials**

# **Title: Unraveling the microbial interactions and metabolic potentials in pre- and post-treated sludge from a wastewater treatment plant using metagenomic studies**

**Running title: Metagenomes of wastewater treatment plant**

**Authors:** Chandni Sidhu^1^, Surendra Vikram^1,2^, Anil KumarPinnaka^1*^

**Affiliation:** 1. Microbial Type Culture Collection & Gene Bank (MTCC), CSIR-Institute of Microbial Technology, Chandigarh-160036, India

1. Centre for Microbial Ecology and Genomics, University of Pretoria, Pretoria 0028, South Africa

**Corresponding Author**

*Dr. Anil Kumar Pinnaka

MTCC-Microbial Type Culture Collection & Gene Bank,

CSIR-Institute of Microbial Technology,

Chandigarh-160036, India

E-mail: apinnaka@imtech.res.in

**Supplementary Table S1:** General features of WWTP, Raipur Kalan, as adopted from (Shar*ma et a*l., 2013)

| Parameters | Influent | Effluent |
| --- | --- | --- |
| pH | 7.2 | 8.1 |
| BOD | 166.3 mg/L | 33.6 mg/L |
| COD | 338.3 mg/L | 148.3 mg/L |
| NO_3_-N | 3.1 mg/L | 1.9 mg/L |
| NH_3_-N | 25.9 mg/L | 32.4 mg/L |
| PO_4_^‑^ | 15.3 mg/L | 4.8 mg/L |

**Supplementary Table S2:** Sequence data obtained after performing quality filter parameters.

| S.No. | Features | RS | DS |
| --- | --- | --- | --- |
| 1. | Basepair count | 932,831,892 | 1,152,853,078 |
| 2. | Sequence count | 5,229,283 | 6,408,171 |
| 3. | Mean Sequence length | 176±58 bp | 179±59 bp |
| 4. | Mean GC% | 44±10% | 48±10% |
| 5. | Artificial duplicate reads | 311,655 | 633,846 |
| 6. | Predicted Protein features | 3,225,139 | 3,899,457 |
| 7. | Predicted rRNA features | 674,488 | 779,323 |
| 8. | Identified Protein features | 895,850 | 919,708 |
| 9. | Identified rRNA features | 5,590 | 4,780 |
| 10. | Identified Functional categories | 668,348 | 704,245 |

**Supplementary Figure S1:** Class level taxonomy comparison of both RS and DS using **(A)** MEGAN v5.10.6. **(B)** Metaxa

**
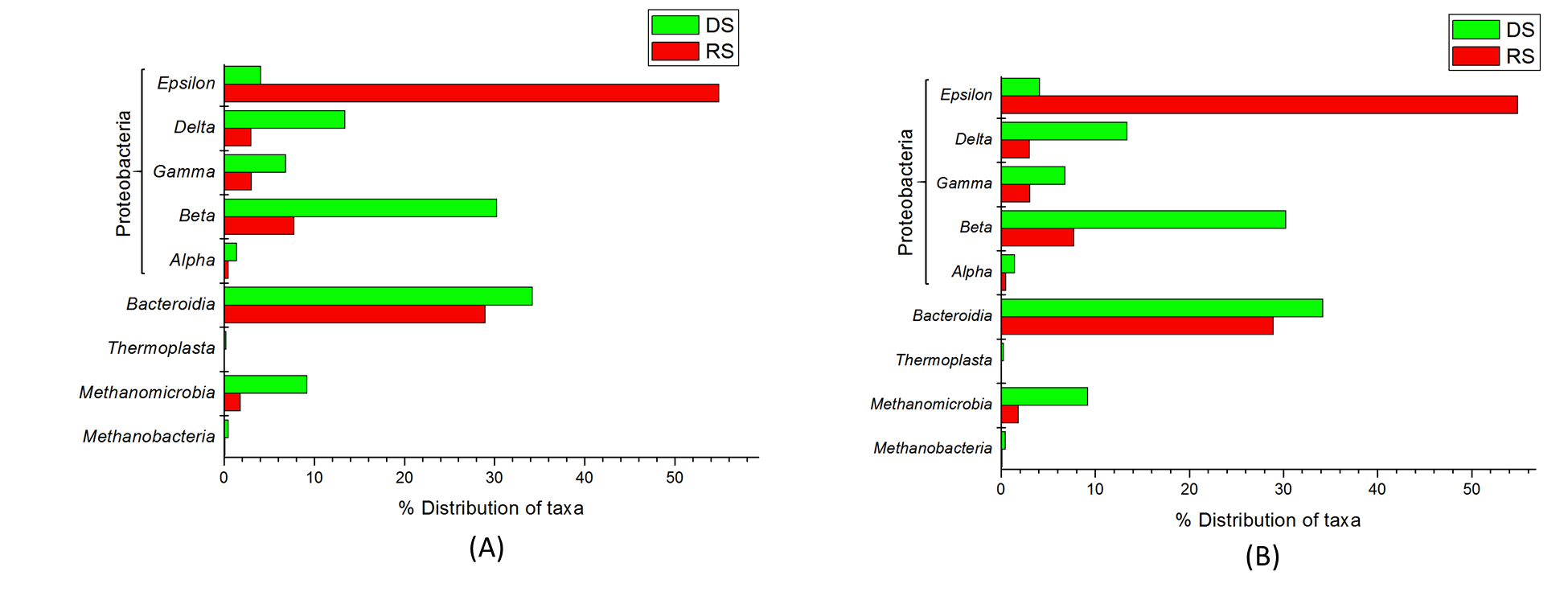
**

**Supplementary Figure S2:** Division of community structure at species level in RS and DS. Significance was determined using two-sided Fischer’s exact test with a CI (Confidence interval) of 0.95 and Storey FDR with the corrected *p*-value <0.05 was used to correct the multiple comparisons.


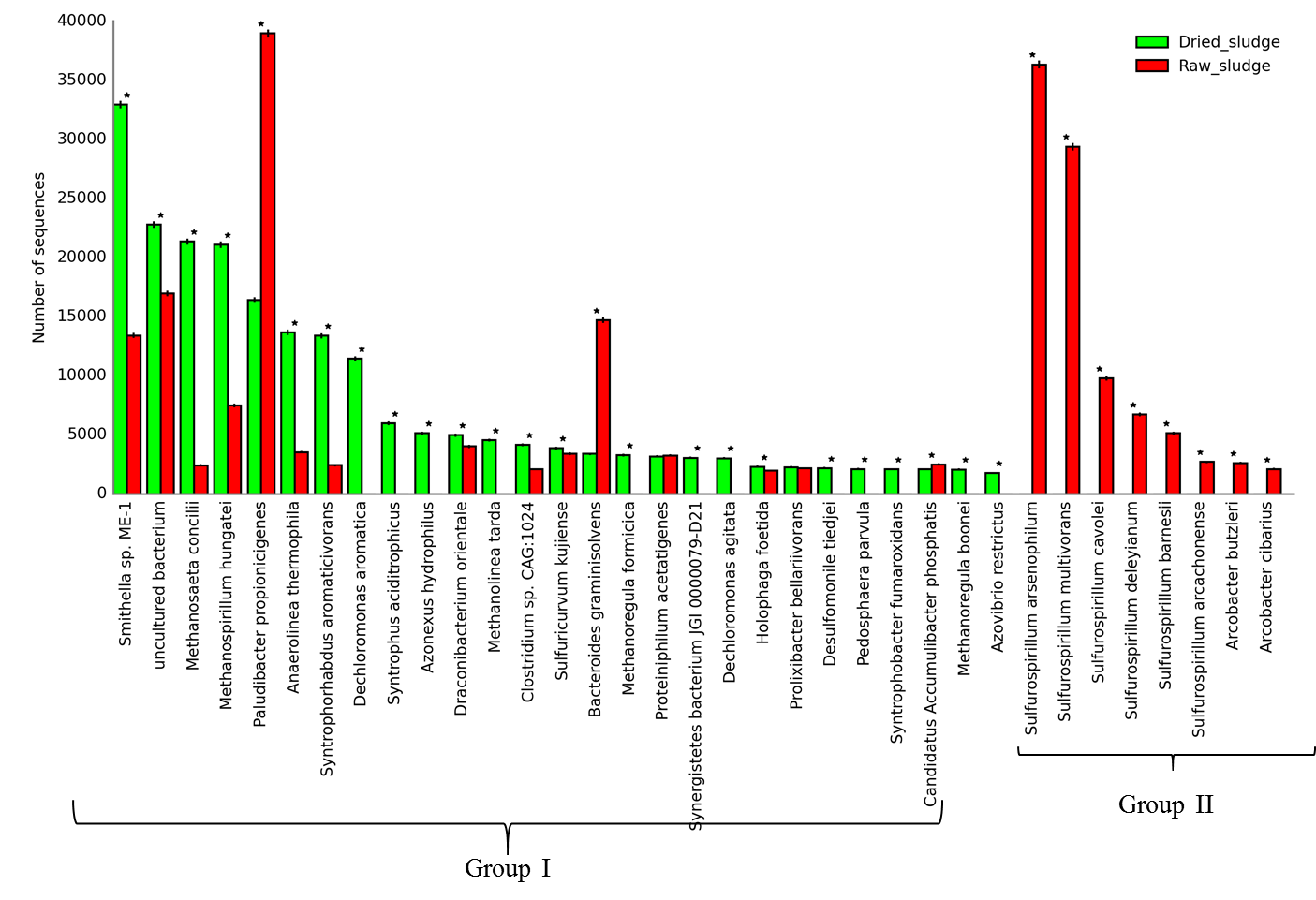


**Supplementary Figure S3: (A)** Evolutionary relationships of taxa-The evolutionary history was inferred using the Neighbor-Joining method. The percentage of replicate trees in which the associated taxa clustered together in the bootstrap test (500 replicates) are shown next to the branches . The tree is drawn to scale, with branch lengths in the same units as those of the evolutionary distances used to infer the phylogenetic tree. The evolutionary distances were computed using the Maximum Composite Likelihood method and are in the units of the number of base substitutions per site. The analysis involved 40 nucleotide sequences. There was a total of 353 positions in the final dataset. Evolutionary analyses were conducted in MEGA6 . Sequence annotation was done using EzTaxon (Chun *et al*., 2007). (**B)** Comparison of methanogenic diversity elucidated from both the amplified library and shotgun library clones.


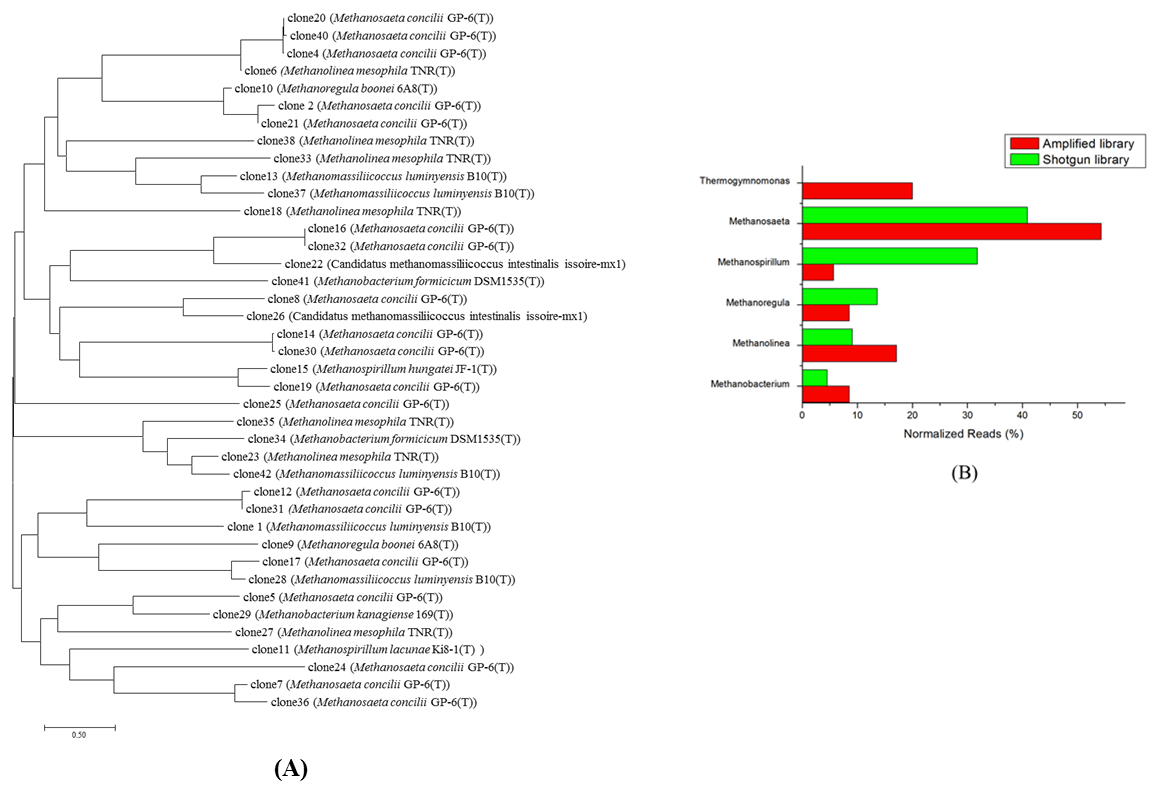


**Supplementary Figure S4:** Classification of pathways related to KEGG analysis of degradation of xenobiotics using MEGAN v5.10.6.


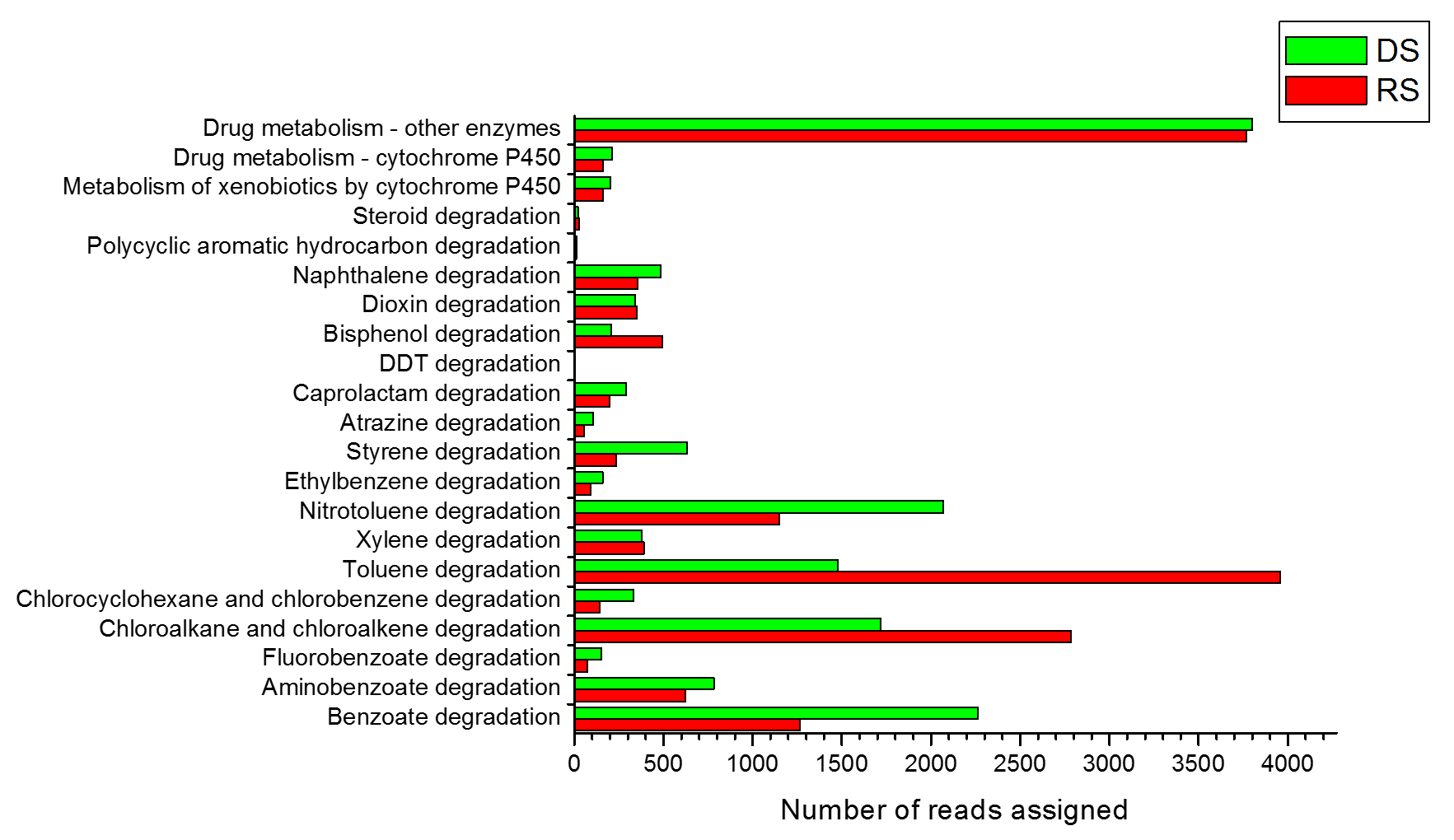


**References**

Chun, J., Lee, J., Jung, Y., Kim, M., Kim, Seil., Kwon K., Byung., Lim. and Young-Woon (2007). EzTaxon: a web-based tool for the identification of prokaryotes based on 16S ribosomal RNA gene sequences. *Int. J. Syst. Evol. Microbiol*. 57, 2259–2261. doi: [10.1099/ijs.0.64915-0](http://dx.doi.org/10.1099/ijs.0.64915-0)

Felsenstein, J. (1985). Confidence Limits on Phylogenies: An Approach Using the Bootstrap. *Evolution* 39, 783-791. doi: 10.2307/2408678.

Sharma, P., Khitoliya, R., and Kumar, S. (2013). A Comparative Study of Sewerage Treatment Plants With Different Technologies In The Vicinity Of Chandigarh City. *IOSR J. Environ. Toxicol. Food Technol.* 4, 113-121.

Tamura, K., Nei, M., and Kumar, S. (2004). Prospects for inferring very large phylogenies by using the neighbor-joining method. *Proc. Natl. Acad. Sci. U.S.A.* 101, 11030-11035. doi: 10.1073/pnas.0404206101.

Tamura, K., Stecher, G., Peterson, D., Filipski, A., and Kumar, S. (2013). MEGA6: Molecular Evolutionary Genetics Analysis version 6.0. *Mol. Biol. Evol.* 30, 2725-2729. doi: 10.1093/molbev/mst197.
